# Abstracts and Gleanings

**Published:** 1884-04-20

**Authors:** 


### ABSTRACTS AND GLEANINGS.

**Tongue in Health and Disease.**—Dr. A. W. Wallace condenses in the Midland Medical Miscellany Dr. Beale's remarks on the tongue, as contained in his admirable little work on slight ailments. A healthy tongue is best known by negative characters, rather than by what it is. In order, therefore, to define the healthy tongue, the following conditions are to be excluded: First, the creamy, white tongue, which denotes unremoved epithelium and metabolism of tissue in abeyance. Second, the furred tongue in which the papillæ are elongated, and to which the epithelium adheres in long threads; this tongue is characteristic of inflammation. Third, the pale, sodden tooth-marked tongue, which is indicative of anæmia. Fourth, the red tongue with enlarged papillæ, as seen in the "strawberry tongue" of scarlatina; the smooth and glazed, as in the "irritable tongue," which corresponds to the irritated mucous membrane elsewhere, as in the lungs from phthisis, or intestines from diarrhœa. Fifth, the dry, brown tongue, pathognomonic of the typhoid state, in which blood exudes and dries on its surface, the secretion of saliva being nil. Sixth, the aphthous tongue, which is often followed by "punched out" ulcers. This condition of the tongue is not particularly significant of any constitutional disturbance. It is more properly a local affection, and is to be treated with the chlorate of potash. And seventh, the red, fissured tongue, which is generally called syphilitic. Dr. Beale, however, says that this is not necessarily syphilitic, although he has found that the exhibition of the iodide of potassium, with or without small (1-32 of a grain) doses of biniodide of mercury, is the most successful treatment.—*Investigator.*

**Rule for Reducing Dislocations of the Hip Joint.**—Having flexed the leg on the thigh, and the thigh on the pelvis, slowly rotate the limb as far as possible, inwards or outwards, according as the toes pointed in or out before beginning the manipulation; then rapidly and forcibly rotate the limb in the opposite direction, and the head of the femur will usually slip into the acetabulum.

For example: In the iliac and the sciatic dislocations, the toes point inwards; therefore, rotate inwards as far as possible, and afterwards rotate outwards. In the pubic and thyroid dislocations the toes point outwards; hence, rotate the limb outwards still more, and then inwards.—*The Polyclinic.*

**Intestinal Obstruction Cured by Capillary Entero-Puncture.**—Dr. Giulio Dozzi relates the case of an old woman, aged seventy, who, after eating a large quantity of watermelon and swallowing the seeds, suffered from obstruction of the bowels. Purgatives and injections had been tried with no relief. The meteorism was enormous. He determined to try entero-puncture, using trocar No. 2 of Dieulafoy's aspirator. Four punctures were made, two in the right iliac region, the third in the left upper fourth, and the fourth in the left lower fourth. From three punctures issued an immense quantity of gas: from the fourth no gas, the trocar being plugged with fæcal matter. A dose of oil given the same evening procured four copious evacuations, and the patient made a good recovery. One of the punctures gave rise to a small abscess. In this case, peristaltic action was evidently prevented by the enormous quantity of gas, arising from the decomposition of the retained fæces.—*Gaillard's Medical Journal.*

**A New Anæsthetic, Bromoform.**—At a meeting of the Society of Physicians of Vienna Dr. Caj. Freiherr v. Horroch reported the results of some experiments with bromoform.

The bromoform was discovered by Lowig in 1832. It is prepared from the hydrate of bromal, by the addition of an alkali. It is an oily, colorless liquid with a pungent, ethereal and aromatic odor and a sweetish taste. It boils at 151° C., is soluble in warm water and ether.

The physiological action of bromal was first tested by Dr. S. Steinauer (Virchow's Archiv, May 19, 1870), who found that it relieved pain and produced sleep. He attributed the action in part to the formation of bromoform. The action of bromoform, itself, was tested by Dr. Horroch (Allgemeiner Medicin. Zeit., No. 3, 1884) in three series of experiments—first, by inhalation; second, by hypodermic injection; third, by giving it by the mouth.

In the inhalation experiments it was found that narcosis was easily produced in animals, the anæsthesia being complete, and the excitation stage short and of a mild form. After recovering from the anæsthesia the animals did not vomit and appeared well.

Hypodermic injections of one gramme of bromoform produced in large animals complete narcosis, lasting for an hour. During this time the temperature fell from 2° to 4° C.

The internal administration of bromoform in doses of one to one and one-half grammes produced sleep of several hours' duration.

The animals experimented upon were rabbits and cats, and the physiological effects upon these animals may be summed up as follows: The respiration is not notably affected. The pulse continues strong, regular and of ordinary frequency. The peripheral ends of the vagus retain their irritability. The blood-pressure is diminished. Reflex excitability is entirely abolished, and irritation of the sciatic nerve failed to effect any changes in blood-pressure. In very profound narcosis the cortical brain-centres lost their irritability. The temperature of the animals sank in dogs 3.5° C., in rabbits about 4.5° C.

As to the value of bromoform as an anæsthetic in surgery, four experiments had been made upon adults in Professor Albert's clinic. Three men had been successfully anæsthetized. In these cases the excitation period had been short: the patients did not cry or struggle. The stomach was not disturbed. Children had been easily anæsthetized.

On the other hand, bromoform, as an anæsthetic, appeared to be milder than chloroform, and its vapors were somewhat irritating to the respiratory passages.—*Med. Rec.*

**The Treatment of Hay Fever.**—Mr. W. F. Phillips, of St. Mary Bourne, Andover, writes: It is just over five weeks since a lady placed herself under my care for the treatment of hay-fever, or summer catarrh—a very much better name. She had suffered severely for many years, and sometimes from the end of May to near the end of July, with little or no intermission, unless she kept indoors. Her mother, it is worthy of remark, was very sensitive to the odor of certain flowers, and was affected by some of them even to the extent of fainting. She was not subject, however, to summer catarrh.

Knowing how exceedingly unsatisfactory is the treatment recommended and practiced for this disease, as is sufficiently evident from the recent communications to the Journal on the subject, I sought for rational indications that might guide me to the selection of a remedy. I thought of the neurosis that seems to underlie most cases of this kind, and to constitute the essential cause or predisposition on which the disease depends; of the characteristic symptoms of the malady; the injection of the conjunctiva; the hyperæmia and hyperæsthesia of the nasal cavities; the excessive secretion of tears and mucus; and then I bethought me of a drug whose physiological action might indicate the possession of the power to control such symptoms. Belladonna was the drug that suggested itself at once, and I determined to give it a trial, all the more hopefully because I remembered how strikingly useful on similar indications, and by a parity of reasoning, I had often found it in ordinary conjunctivitis and simple catarrh. I began with the following prescription:

℞ Succi belladonnæ........................... m xxiv,

Aquam ad........... ....................... ℥ iij.

Misce.

A teaspoonful to be taken every hour till relief is obtained. The medicine was taken without the production of any undesirable effect, and with very marked advantage indeed—an advantage that became still more evident and unmistakable, both to the patient and myself, when the dose was increased from one minim to one and a quarter (half a drachm in three ounces). Once, too, when the eyelids were especially tender, the patient was advised to use the mixture as a lotion to the affected parts, and this local application was found to be a most useful addition to the internal administration of the remedy. Repeatedly, when the symptoms of an attack had been allowed to begin, the patient found prompt relief after a few doses of the drug; the catarrhal affection disappearing first, and then the asthmatic; and on taking it regularly every day after the malady had been subdued, she has found, to her delight, that she can take her walks abroad through blooming grass and flowers without the least protection or precaution—a thing she had not been able to do before for years.

The patient, remembering, no doubt, the failure of past treatment, pronounces the remedy "a great success;" but, however satisfactory the case may be, it is, as far as I know, a solitary one, and, therefore, stands in need of confirmation and support.—*Brit. Med. Jour.*—*Chicago Med. Jour.*

**Locality of Malaria.**—We now proceed, says J. W. Dowling, M.D., in the Medical Investigator, to consider the peculiarities of malaria as to locality. Bartlett says: "With certain limited exceptions, it may be said to encircle the earth in a broad belt parallel with the equator; its northern and southern boundaries quite irregular in their disposition—now approaching to the line of the tropics, and now receding from it. The portions of this immense territory which are entirely exempt from periodical fever increase with the distance from the equator; while within the tropics and within the range of several degrees beyond them, these portions are confined mostly to geological formations, and to elevated situations. The particular regions most extensively and constantly the seat of malarial disease in its most malignant forms are low and wet lands situated in hot climates and covered with a rank and spontaneous vegetation—the flat wooded sea-coast, the interior swamps and marshes, and the rich alluvium of the deltas, and courses of the great rivers."

The poison known as malaria seems to emanate from the earth. It floats in the atmosphere, hovering near the surface of the earth, and undoubted evidence exists of its being carried by the wind from malarial spots to localities which would otherwise be entirely exempt from it—to a certain extent purifying the regions where it is generated.

It is more apt to be found in localities where the upper crust of the earth is moist and subjected to the action of the heat of the sun. In warm climates, wherever there is a subsoil of clay, or a rocky bed covered with a thin layer of soil, malarial diseases are almost surely to be found.

As a rule, wherever is found a fog which hovers but a few feet from the earth's surface, making its appearance with the setting of the sun, and gradually disappearing with its rising, malaria is sure to be present.

It is found also in localities entirely free from moisture, in some sandy regions, sandy to the depth of many feet, where there is no vegetation, but this is the exception.

It is affected by the temperature of the atmosphere—frost rendering it entirely inactive. Trustworthy authorities assert that after once being submitted to an atmosphere of or below the freezing point, it is not again operative in producing disease till it has been exposed to a temperature above sixty degrees Fahrenheit.

It is more active at night than during the day. Evidence has accumulated to prove that certain localities exceedinly dangerous at night are perfectly healthful during the day. Watson records an instance: "In 1836 the Phœnix, ship of war, was returning from the coast of Guinea. The officers and ship's company were perfectly healthy till they touched at the Island of St. Thomas. Here nearly all of them went on shore; sixteen of the number remained for several nights on the Island. Every one of them contracted the disorder, and thirteen of the sixteen died. The rest of the crew, consisting of 280 men, went in parties of twenty or thirty on shore in the day, and rambled about the island, hunting, shooting and so on; but they returned to the ship at night, and not one of those who so returned suffered the slightest indisposition. Exactly similar events occurred the following year with the same ship at the same place, when she lost eight men out of ten who had imprudently remained all night on shore, while the rest of the ship's company, who, after spending the greater part of the day on shore, always returned to their vessel before night, continued in perfect health." These are but two of many examples of the same kind.

The fact that those remaining on ship-board did not contract the disease proves one of two things, viz., that the wind was not blowing from the land, or that water has the power of absorbing the malarial poison.

It has been conclusively proven that miasmatic atmosphere passing even over a small body of water becomes inoperative. The poison, hovering, as it does, near the surface, is undoubtedly absorbed by the water. Proof positive exists that water which has absorbed large quantities of malarial poison can impart intermittent fever to those drinking of the water so impregnated.—*Med. Investigator.*

**How Typhoid is Spread.**—An item in one of the New York papers states that a little local epidemic of typhoid fever in New Jersey had been traced back to the dairy of one of the farmers who supplied the town with milk. A case of typhoid in his house had been distributed in his milk throughout the entire neighborhood. It is not always that suspicions of the agency of milk in such cases are proved good. A notable case in point is the epidemic of last year in the upper part of New York Island, at Carmansville. This was at first attributed to one of the milk dealers, who was in consequence nearly ruined. But a thorough inspection by the Board of Health, who were at first completely baffled, finally traced the disease to an accumulation of water in the end of a sewer which had no outlet. The opening of the sewer ended the fever.

A case of more than usual interest in sanitary detection is reported by the Pall Mall Gazette. During August last the number of cases in St. Pancras Vestry, London, which usually has an average of 40 to 45, rose to 223, and the disease spread until 431 persons had been attacked, and 92 died. The sanitary police were called in. A map of the track of the epidemic showed that it was independent of the drinking-water, of the plumbing and sewerage, and of the locality. It was then found out that of the 431 who had been taken down, 368 obtained their milk from one man. Wherever this man's milk carts went the fever followed.

An examination of the premises of the suspected dealer was made without revealing the slightest cause for the origin of the typhoid there. But when his sources of milk supply were investigated, it was found that the houses that were infected had all been taking milk which he had procured from one place. a farm at St. Albans. One step further was gained when it was found that several of the railroad porters who had—as railroad porters will do when they have a chance—been taking drinks on the sly out of this St. Albans milk had come down with typhoid. The farm was visited, and there was the fever raging among the residents, and being distributed from them to the distant homes in London.

The theory of the medical officer who performed this clever bit of detective work, as to the way in which the fever originated on the farm itself, is interesting. He noticed that the well and the cesspit of the farm were within twenty feet of each other. A sycamore tree overhangs both well and cesspool. Its roots spread as far underground as its branches overhead, and along them water could easily percolate from the pit to the well. The milk cans were washed in the water from the well, and in that way caught the contagion of the deadly filth which they transmitted through the milk into the blood of London.

The increase of typhoid in this country is probably an indication that we are losing the comparative immunity which the sparseness of our population and the newness of the country have hitherto given us from the contamination of our water supply by recklessness like that of the St. Albans farmer, which is as frequent in this country as in England.—*Eclectic Med. Journal.*

**Treatment of Nævus.**—A writer in the British Medical Journal says: Since 1861 I have treated all nævi projecting more than a sixteenth part of an inch from the surface in this manner:

The little operation is very simple. Wood's syringe, with a very fine needle, is the only instrument required. Sufficient tincture of iodine having been drawn into the syringe to fill the nævus, the needle is introduced through the skin at about a line from the circumference of the nævus, and passed to its center. The piston is propelled slowly home, so as to force the tincture into every part of the growth. This is facilitated by moving the point of the needle into every part of the nævus. On withdrawing the instrument pressure is made on the small punture for a few seconds, and the proceeding is complete.

I have practiced this treatment many times since the year 1861 with complete success. One injection generally succeeds; sometimes several are required. Usually a slight vesication occurs on the surface of the nævus, then a white spot or spots appear, which spread in all directions until the vessels are obliterated; a slight depression of surface alone remains. In two cases the action was more violent and more rapidly curative.

In a little boy who had a small thick nævus in the inside of the upper lip, five minims induced a sloughing of the growth, which shelled out, leaving a fine cicatrix. In a little girl, a nævus of the size of a filbert, situated in the left labium, sloughed and shelled out in a similar manner. In these two cases the abnormal structures could not bear even the gentle stimulus of the tincture of iodine, and so lost their vitality.

**Vaseline Cerate, a Convenient Basis for Ointments Intended for Applications to the Eyelids.**—Dr. Theobald, of Baltimore, read a brief paper on this subject, in which he stated that he had been using a cerate made of yellow wax and vaseline, with much satisfaction, for several months, both in private and hospital practice, as a basis for ointments intended for application to the eyelids. It was made by melting the wax and vaseline together with a gentle heat, and stirring the mixture until it hardened, combining one part of yellow wax with four of vaseline, which proportion gave the ointment sufficient firmness, except, perhaps, in very hot weather, when the proportion of wax might be increased from one to three. Dr. Theobald also exhibited a specimen of ointment of the yellow oxide of mercury, containing two grains to one drachm of vaseline cerate, which, though prepared nearly four months since, and kept with no special care, still retained its bright-yellow color, and had undergone no appreciable change. He had been told that cerates prepared with vaseline were in use, but was not aware that attention had been called to the convenience of employing them in the manner suggested.—*New York Med. Jour.*

**Rattle-snake Poisoning Treated by Potassium Permanganate.**—Emil Bories, a student of medicine in Bellevue, reports the following in the Polyclinic: In the early part of May, 1883, the weather being very warm, a man, aged about thirty-five, was bitten in the calf of the right leg by a rattle-snake. He was stepping close to some loose rocks and heard the peculiar warning rattle of the snake, but, before he could recover himself, was struck. He was taken to his hut by his companion, who first killed the reptile and secured its rattles, which numbered seven, with the button. By the time I reached the patient, four hours had elapsed. I found him very pale, cold sweat covering his face and extremities, pulse slow and irregular. He complained that his sight was failing, and was in great pain. He had vomited several times. I examined the wound and found the leg badly swollen, presenting a very glossy surface of an erysipelatous nature, quite tender to the touch. The point of entrance of the fang was quite pale, while the surrounding surface was of a light red hue. I asked for stimulants, but none could be had. I had read that potassium permanganate had been used with happy results in cobra poisoning; I thought it would be well to try it here. I immediately injected about one-fourth of a grain into the wound and the same quantity into the arm, for fear that the solution injected into the wound would not be taken up into the circulation. I also gave him two grains by the mouth. Before leaving him I arranged matters so that he could receive two grains by the mouth and one-fourth of a grain injected every hour, alternately, but I had little hope for success. I was, indeed, much surprised, on visiting him next morning, to find him looking well, his pulse full and regular, his eyesight good, his appetite fair, and the swelling diminished nearly one-half. However, I made a poultice of meal, mixed with a small amount of aqua ammoniæ, and told him to take half a dram of aromatic spirit of ammonia every three hours, continuing the use of the potassium permanganate by the mouth, but discontinuing the injection. On the following day he was much better. I then discontinued all but the permanganate, which was to be taken every three hours, keeping the wound moist with about ten per cent. solution of ammonia water. The fourth day he was out and at work of a light nature.—*Med. Herald.*

**A New Form of Eye Bandage.**—Peschel (Arch. f. Augenheilk., xii, 4) has invented a new variety of bandage for the purpose of applying a continous bath to the eye, hoping thus to obtain as complete disinfection as possible. He has a sort of rubber mask constructed, which can be applied, water-tight, to the face below the eyes, against the temples, and over the nose, by means of a flexible metallic bow or arch, and an air-bag beneath it. This mask is open upward toward the forehead, and in a sitting posture the patient can thus keep the eyes continually immersed. This mask-bandage is constructed of three shapes: for the right eye, for the left eye, and for both eyes. Since September, 1882, Peschel has employed the apparatus in a great many cases, partly to destroy certain organisms already present, and partly to prevent their introduction from without; and the results have been most satisfactory. —*N. Y. Med. Record.*

**Horrible Hiccough Cured.**—Doctor Ruhdorfer, in the Allgemeine Wiener Medizin. Zeitung, No. 38, reports a case of hiccough lasting three months, which morphia, hypodermically, could only check for a few hours or days. It resisted all the usual remedies. The patient dragged on three months under various remedies, morphia being administered whenever an attack lasted beyond eight hours. But at last the attacks became overpowering, and the hiccough was so loud that the patient could be heard outside the house, through two doors. She sat up in bed, supported by her parents; there were dyspnea and cyanosis, and the head was jerked in all directions; the pulse was small and frequent; the neck was distended. Remembering a case in the Revue Medico-Chirurgicale, Dr. Ruhdorfer injected a solution of pilocarpine hydrochlorate (three centigrams in a gram of water.) The hiccough was at once cured, as if by magic, and has never returned since.

**Prof. Parvin** says he believes Negroes to be very fond of musk, and asked for investigation on this point by members of the class. A pharmacist of very large experience in the South writes that he once kept a record of perfumes sold to Negroes, and that nine-tenths of them bought musk, and the other tenth citronelle, the latter being sold to the lowest class. He says they are excellent judges of musk in pods, and are hard to deceive.—*Col. & Clin. Record.*

[The musk of the Negro is native to the animal himself.—*Ed. Record.*

**Flatulence.**—In flatulence, Dr. Bruen prescribes a pill containing five grains of bicarbonate of soda and five drops of oil of eucalyptus two hours after meals. Pepsin or pancreatin with milk food and the mineral acids with meats should be directed to be taken immediately after meals.—*Med. Summary.*

**Sciatica and Lumbago—Injection of Sulphuric Ether.**—Mr. J. B. James, in the British Medical Journal, says: The successful results I have invariably found attending this system of treatment, which I have adopted for the last four years with my rheumatic patients, have decided me to give publicity to the course from which I have seen so much benefit derived. Its plan is simple enough. After preliminary dry cupping over the seat of lesion, I inject subcutaneously ten minims of sulphuric ether, gradually increasing it till I have injected thirty minims (assuming I find no marked progress in the course of a week of treatment above named.) I have found it advisable to precede this by a brisk purgative at the outset, and to administer a mixture containing five grains of salicylate of soda in an ounce of infusion of gentian every two hours, concurrently with internal and external applications. In not one case have I yet found this curative system fail; but in about a week's time, usually, the patient is cured. Sufferers from lumbago have come to me nearly bent double with pains in the lumbar region, and have walked away erect and free from their distresses after dry-cupping. I have seen sciatic patients come in limping, and go out free from the least indication.

I can especially instance the case of one patient, an old man, who had been the round of all the London hospitals. to no avail, for nine years previous to his consulting me. He had been given up by all as a hopeless case. On my asking whether he was willing I should try a method of treatment on lines hitherto unattempted, and on his consenting to the same, I pursued the subcutaneous injection already described until I attained the administration of a drachm of sulphuric ether. Marked improvement followed this course, which, however, I was obliged to suspend, owing to the formation of a hard cicatrix over the seat of the sciatic nerve. Nevertheless, after this was removed, the patient ultimately found himself completely recovered, and during the five years which have elapsed since he first came to me, he has sustained but one attack of sciatica of a very slight character.

I sincerely hope these few remarks on my own practical experience of this system of treatment of cases, the persistency of which so frequently baffles the efforts of the most experienced practitioners, may prove of some practical utility in similar cases. —*Brit. Med. Jour.*

**Ferry-boat Amenities.**—A Chinaman came into the ladies' cabin on the ferry boat and took a seat beside an Irish market woman. He seemed to want to make himself agreeable, and, rubbing his hands, remarked: "Belly cold." The woman looked at him with an air of contempt and replied: "If you'd put your shurt inside yeur pants, your belly wouldn't be cowld, you haythen blaggard."—*Ex.*

**A Boarder** complained that things were too slow around the house, and informed the landlady that he would like to dynamite earlier.

**Prof. S. W. Gross** (College Record) thinks that carbolic acid, in any safe solution, is not active enough to kill germs. For the antisepsis of opened lumbar abscesses he recommends the bichloride of mercury solution, one part in a thousand.

**Novel Mode of Bleeding.**—The latest mode of blood-letting is given in a late number of the British Medical Journal by Dr. Charles Coppinger, who relieved a serious cerebral congestion by introducing the aspirator needle into the external jugular vein and abstracting at first four ounces of blood, and half an hour later six ounces more. The patient was a fat and plethoric lady fifty years of age.—*Jour. Amer. Med. Assoc'n.*

**To Prevent Abscess.**—Dr. Makune (Brit. Med. Jour.) says: "In cases of threatening abscess I have found a combination of iodide of potassium with cinchona a valuable remedy. I may say that Indian women use the fresh leaves of datura and a species of stramonium as antigalactagogues, and castor-oil plant leaves as a galactagogue.

**Effect of Pilocarpin upon the Hair.**—Dr. Prentiss (Louisville Med. News) says: I have had one decided case in which persistent local application of pilocarpin has stimulated the growth of the hair in alopecia.

As to change of color, several circumstances must combine to produce that effect, namely: (1) The hair must already be light or blonde; (2) the medicine must be given in full doses; (3) it must be long-continued.

Evidently, by referring to the effects produced by the medicine in my case, no one is likely to use it for the purpose of coloring the hair, and most cases that call for such use of the remedy as a medicine are fatal in a short period.

**Death from the Self-Administration of Chloroform.**—Another death has again shown the fatal danger which attends the self-administration of chloroform. Last week an inquest was held respecting the death of Walter Woodcock Wilmot, a medical student, aged eighteen years, who was found dead in bed in his father's house, with a bottle containing chloroform in his right hand. Evidence showed that death was due to the inhalation of chloroform, and that the deceased had long been in the habit of employing the anæsthetic for the purpose of inducing sleep.—*Brit. Med. Jour.*

**Asthma.**—Dr. Greensword (in Med. Summary) says: Nearly twenty years ago a friend suggested the use of one part of the oil of peppermint to three of alcohol as a substitute for the belladonna, and I have used it successfully ever since. I afterwards thought that it would, if inhaled, take the place of the fumes of the nitrate of potash, or the burned paper saturated in that salt, so highly prized by some, and I find it does so, and does the work better than the nitrate of potash. This same treatment is also excellent in pleurisy.

